# Motor Fault Diagnosis Based on Convolutional Block Attention Module-Xception Lightweight Neural Network

**DOI:** 10.3390/e26090810

**Published:** 2024-09-23

**Authors:** Fengyun Xie, Qiuyang Fan, Gang Li, Yang Wang, Enguang Sun, Shengtong Zhou

**Affiliations:** 1 School of Mechanical Electrical and Vehicle Engineering, East China Jiaotong University, Nanchang 330013, China; fqy18532671715@163.com (Q.F.); wwyy0130@163.com (Y.W.); sngzm999@163.com (E.S.); zhoust@ecjtu.edu.cn (S.Z.); 2 State Key Laboratory of Performance Monitoring Protecting of Rail Transit Infrastructure, East China Jiaotong University, Nanchang 330013, China; 3 Life-Cycle Technology Innovation Center of Intelligent Transportation Equipment, Nanchang 330013, China; 4 School of New Energy, Ningbo University of Technology, Ningbo 315211, China; 2021038080400012@ecjtu.edu.cn

**Keywords:** motor fault diagnosis, vibration signals, neural networks, deep learning, convolutional block attention module

## Abstract

Electric motors play a crucial role in self-driving vehicles. Therefore, fault diagnosis in motors is important for ensuring the safety and reliability of vehicles. In order to improve fault detection performance, this paper proposes a motor fault diagnosis method based on vibration signals. Firstly, the vibration signals of each operating state of the motor at different frequencies are measured with vibration sensors. Secondly, the characteristic of Gram image coding is used to realize the coding of time domain information, and the one-dimensional vibration signals are transformed into grayscale diagrams to highlight their features. Finally, the lightweight neural network Xception is chosen as the main tool, and the attention mechanism Convolutional Block Attention Module (CBAM) is introduced into the model to enforce the importance of the characteristic information of the motor faults and realize their accurate identification. Xception is a type of convolutional neural network; its lightweight design maintains excellent performance while significantly reducing the model’s order of magnitude. Without affecting the computational complexity and accuracy of the network, the CBAM attention mechanism is added, and Gram’s corner field is combined with the improved lightweight neural network. The experimental results show that this model achieves a better recognition effect and faster iteration speed compared with the traditional Convolutional Neural Network (CNN), ResNet, and Xception networks.

## 1. Introduction

As an important innovation guiding the future of transportation, autonomous driving technology has gradually become the focus of global technological development [1]. However, in order to realize the safety, efficiency, and reliability of autonomous driving, diagnoses of the health status and faults of vehicles are particularly important. According to research, vehicle failure is one of the main causes of traffic accidents [2]. Once a vehicle suffers an electric motor failure, its driving performance will be significantly reduced, which may lead to unstable driving and thus increase the risk of accidents [3]. Therefore, fault detection in electric motors is crucial for self-driving vehicles.

Three-phase induction motors are the backbone of the industrial sector and play a vital role in fields such as power generation, transportation, and manufacturing. Their primary applications include electric vehicles, fans, compressors, and blowers. Induction motors are often subjected to poor lubrication, high temperatures, high pressures, and prolonged exposure to thermal and mechanical stresses, which inevitably lead to unexpected failures. According to a report by the U.S. Electric Power and Energy Research Center (EPRI) [4], common failure types of induction motors include mechanical failures, which account for about 53% of all cases (such as bearing failure, rotor imbalance, and loose parts), and electrical component failures, which account for approximately 47% (such as damage to stator and rotor windings, casting defects in segment bars, and short circuits). Therefore, fault detection of induction motors mainly focuses on stator and rotor faults, with winding faults in the stator and rotor coils being one of the leading causes of motor failure, which forms the basis for this paper. Stator winding failures are often caused by long-term wear and tear, production defects, or oil contamination, leading to insulation failure. Rotor winding failures result from damage caused by a combination of high temperatures, mechanical stresses, and other environmental factors. Among these faults, motor winding turn-to-turn short circuit faults are some of the most common and hazardous. Consequently, monitoring the motor’s operating state and condition is crucial. Common diagnostic methods include general and specialized techniques. The most widely used general diagnostic method is vibration signal detection, which involves time-domain analysis, frequency spectrum analysis, power spectrum analysis, and time-frequency domain analysis using traditional machine learning methods, as well as modern techniques such as deep learning, reinforcement learning, and meta-learning [5].

Data processing has made remarkable progress in the field of fault diagnosis in the past few decades. Bo Wang proposed an open-circuit fault diagnosis technique for residual voltage analysis based on current signals to achieve open-circuit fault diagnosis in permanent magnet synchronous motor drives. Yutong Song proposed a multi-scale feature fusion convolutional neural network fault diagnosis method based on time-domain signals, achieving fault diagnosis in electromechanical actuators [6]. Hong J utilized high-frequency detailed wavelet components to extract the early frequency-domain features of fault signals, achieving electric vehicle lithium-ion battery fault prediction and isolation [7]. However, these studies often only consider the time-domain information or frequency-domain information and neglect the interrelationship between the two, which may lead to incomplete feature extraction and insufficient accuracy.

In recent years, with the rapid development of deep learning technology, more and more studies have applied it in the field of fault diagnosis. For example, Yang T proposed a one-dimensional convolutional spatiotemporal fusion strategy for the intelligent diagnosis of aviation hydraulic piping systems [8]. In vehicles, Gültekin, Ö et al. used the LeNet-5 CNN model as the basis of edge AI to realize the real-time fault detection and condition monitoring of industrial autonomous transit vehicles [9]. Kaplan, Het al. used the LSTM model to realize fault diagnosis in electromechanical conversion chains of electric vehicles [10]. However, some of these deep learning models are too simple to extract features effectively; alternatively, some models are more complex and have very high hardware requirements. The training samples used in deep learning are ever becoming greater, and so it is challenging to keep the model’s complexity within an acceptable range while improving its accuracy. B. Xu et al. used the ESPRIT method to analyze the stator current signal spectrum and applied the Duffing system to further process the results of the ESPRIT spectrum analysis for detecting rotor broken bar faults [11]. Y. Zhang proposed a fusion correlation spectrum method for asynchronous motor fault diagnosis, in which the stator current and vibration signals are fused using correlation spectral features as the diagnostic basis for detecting rotor broken bar faults and stator turn-to-turn short-circuit faults [12].

The introduction of an attention mechanism to further optimize the feature extraction process is an idea worth promoting [13]. Such an attention mechanism can automatically learn the weights of key parts of the signal, enabling the model to pay more attention to the most important information contributing to fault diagnosis [14]. For example, in the squeeze and excitation network (SENet) proposed by Hu, the attention mechanism at play between feature channels is established by modeling the interdependence between feature channels to adaptively recalibrate the features [15]. Wang Y further demonstrated in his paper that the SE network is useful in fault diagnosis in the face of the effectiveness of multi-channel neural networks [16]. However, the SE attention mechanism only considers attention in the channel dimension and cannot capture attention in the spatial dimension. For this reason, WANG published an efficient channel attention mechanism, the Efficient Channel Attention (ECA) module, in CVPR 2020 [17]. Compared to traditional attention methods, it can solve the side effects of dimensionality reduction that occur during model prediction. Some have proposed a multi-target tracking method that introduces a combined efficient channel attention module into the backbone network, which can adaptively extract the most important information from the image and improve the accuracy of target detection.

This demonstrates that combining the attention mechanism can enhance the overall performance of the network, although there have been limited studies combining lightweight networks with attention mechanisms. Therefore, a new method for electric machine fault diagnosis is designed by fusing the CBAM attention mechanism with a lightweight neural network model.

To address the aforementioned challenges, this study adopts a signal analysis approach and first builds an experimental platform for motor fault diagnosis using the most widely deployed three-phase induction motor, with vibration sensors capturing its acceleration and dynamic signals. The time-domain signals are then transformed into a comprehensive Gram angle field map, which improves the holistic capture of motor fault characteristics. The Xception model is employed in this study for pattern recognition. Additionally, by introducing the CBAM attention mechanism, the study aims to further enhance the accuracy of motor fault prediction and detection.

The innovations of this work are the use of the cross-domain fusion image coding method and the new method of the Xception-attention mechanism. We also apply the method to the field of motor fault diagnosis for self-driving vehicles. Through experiments and result analysis, this study expects to verify the effectiveness and advantages of the method and provide new ideas for the solution of motor fault diagnosis in self-driving vehicles.

## 2. Fundamentals

### 2.1. Basic Structure of Convolutional Neural Networks

The basic principle of convolutional neural networks is actually to simulate the visual system of the human brain; based on this, the convolutional layer is used to make the features of the input signals more obvious, and the pooling layer is used to set out a correlation between the local signals and the original signals for the dimensionality reduction operation. In order to further obtain as many signal features as possible, the number of convolutional and pooling layers is usually increased, and then these features are mapped to a one-dimensional fully connected layer and fed into softmax, through which the recognition of the signal is achieved.

In 2012, AlexNet was used in image classification and showed its excellence; this phenomenon attracted the attention of many scholars. Thus, convolutional neural networks began to be used in various fields such as vision, spectroscopy, and images. This network enables the establishment of a model mapping the input to the output. When processing images, the convolutional neural network ultimately needs to compress the data into one dimension through a flat layer and then input them into the softmax layer for classification.

#### 2.1.1. Input Layer

The input layer of a CNN is the first layer in the entire network. Its main role is to receive and process raw data for input into the network [18]. Therefore, the structure of the input layer is crucial for the subsequent recognition of the entire network. It significantly impacts the recognition speed and accuracy of the neural network. The primary functions of the CNN input layer are as follows:

1. When we input the data into the neural network, the input layer receives data that generally consists of vibration, sound, image, and other data. For vibration data, the most common input formats are time series and 2D images.
2. For 2D images (grayscale), the main role of the input layer is to determine the size of the image and the quantity of channel information.
3. For raw data, another role of the input layer is to pre-process the data. For example, this includes normalization (to be input, the image is fixed within a particular range, such as between 0 and 1), standardization (so that the mean value of the image data is 0, the standard deviation is 1), and other types of pre-processing methods. Preprocessing the data ensures the speed of training and the reliable strength of the whole model.

#### 2.1.2. Convolutional Layer

The convolutional layer is the core component of the entire neural network; its main role is to input data through the operation of convolution in order to extract features. It recognizes the patterns and features of the input data by setting the size of the convolution kernel [19]. The set convolution kernel operates on the data to extract the features of the operation; the greater the number of convolution layers that are set, the deeper the convolution, and the greater the number of parameters that will be used, in addition to the fact that deeper features can be obtained.

The formula for the convolutional layer is as follows:

(1) $$x_{j}^{l}=f(\sum_{i\in M_{j}}^{n}x_{i}^{l-1}*k_{i,j}^{l}+b_{j}^{l})$$

Here, $x_{j}^{l}$ is the output; l is the number of layers to be convolved; $x_{i}^{l-1}$ is the input information of the l layer; $k_{i,j}^{l}$ is the weight; $b_{j}^{l}$ is the bias; f(·) is the activation function; $M_{j}$ is the convolution range.

**Figure 1:**
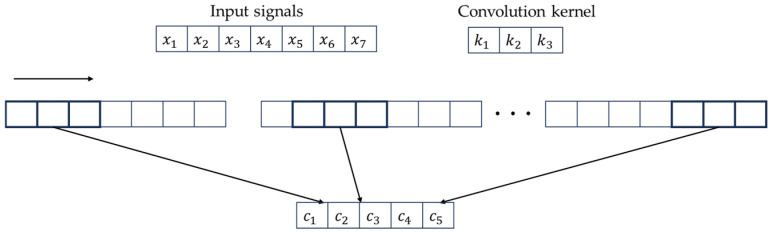
The evolution process of the convolution layer.

For example, for an input signal with a data length of 7, a convolution kernel K of length 3, and a step size of 2, the convolution process of the convolution layer is shown in Figure 1.

#### 2.1.3. Pooling Layer

The pooling layer is another key layer of the convolutional neural network. Its main function is to reduce the spatial size and parameters of the convolved features, which in turn reduces the computational complexity of the whole network model, and this operation is also conducive to the stability of the extracted features. The main parameters of the pooling layer are pool size and stride; different pool sizes correspond to different pooling windows, and the stride is the distance moved on the pooling window [20]. These are the two main parameters of the pooling layer.

The pooling layer process is given by Equation:

(2) $$X_{j}^{l}=f(\beta_{j}^{l}\cdot down(X_{j}^{l-1})+b_{j}^{l})$$

Here, $\beta_{j}^{l}$ is the multiplicative bias; $b_{j}^{l}$ is the additive bias; down(·) is the sampling function.

**Figure 2:**
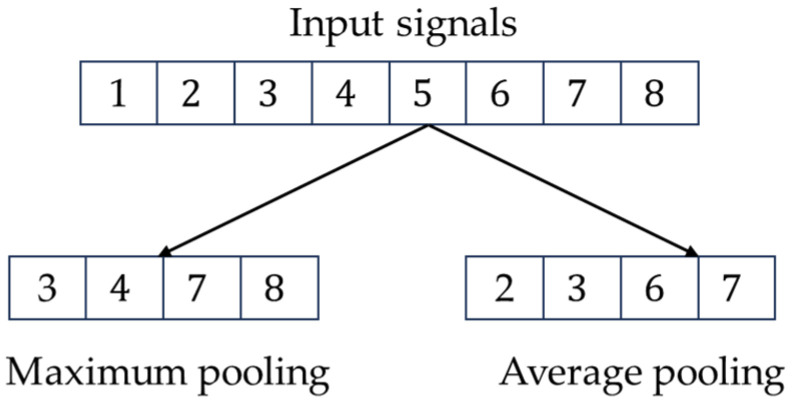
Computational process of the pooling layer.

For example, if the length of the data is 8, the pooling window is 2, and the step size is 2, then the operation process of the pooling layer is shown in Figure 2.

#### 2.1.4. Fully Connected Layer

After convolutional pooling, the convolutional layer is used to extract the features of the original data, while the pooling layer downscales the dimensionality of the features. The fully connected layer connects the neurons throughout the network and links the convolutional network to the softmax layer [21]. This is located at the end of the network, but it transforms the information extracted by the previous convolutional layer, as well as the pooling layer, into the final output for classification and recognition.

**Figure 3:**
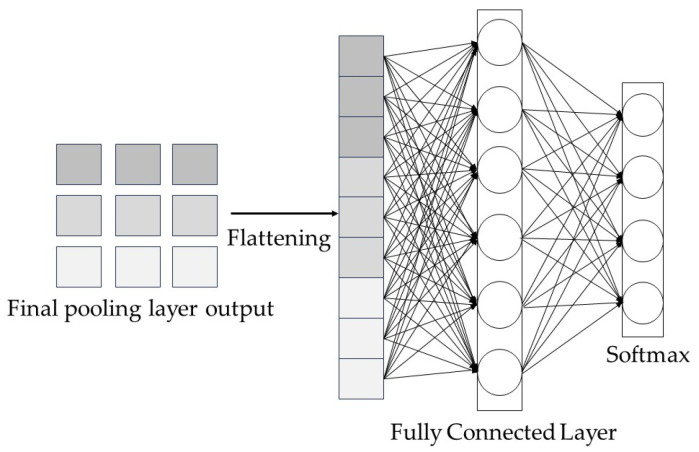
Operational processes in the fully connected layer.

The formula for the forward propagation of the fully connected layer (See Figure 3) is as follows:

(3) $$Z_{j}^{l}=f(\sum_{k=i}^{n}w_{j}^{l}x_{i}^{l}+b_{j}^{l})$$

Here, $w_{j}^{l}$ is the connection weight of the i neurons in layer l to the i neurons in the previous layer; $b_{j}^{l}$ is the bias term of the j neurons in layer l; f(·) is the activation function.

Many scholars have been studying convolutional neural networks increasingly deeply, and the idea of residual learning has been introduced, which solves the previous problem of gradient vanishing, circumvents the deepening of neural networks in one way or another, and thus achieves very good results. The residual neural network can essentially be said to be the framework of the deep convolutional network; the residual structure uses a fast channel, whereby the upper layer of the training features, which does not require too much processing, is directly passed to the next network layer. The ResNet network achieves very good performance compared to other networks; in the training process, it can be applied across the layers of the transfer, and this effectively resolves the degradation of the training process of the network structure.

### 2.2. CBAM Attention Mechanism

#### 2.2.1. Channeling Attention Mechanisms

The steps for the implementation of the channel attention mechanism module in CBAM are as follows: the input feature maps are subjected to global maximum pooling and global average pooling, respectively, and the feature maps are compressed based on two dimensions to obtain two feature descriptions of different dimensions. The pooled feature maps share a multilayer perceptron network, which is first downscaled by 1 × 1 convolution and then upscaled by 1 × 1 convolution. The two feature maps are superimposed and added together, and the weights of each channel of the feature map are normalized with a sigmoid activation function. We then multiply the normalized weights with the input feature map [22].

**Figure 4:**
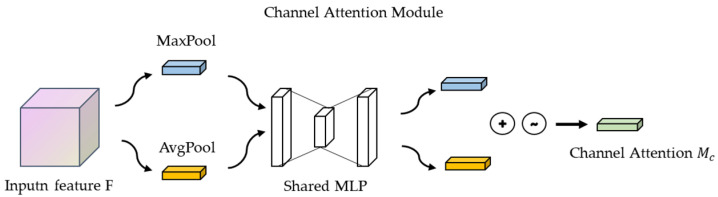
Channel Attention Module.

The output $M_{c}\left(F\right)$ formula for the channel attention module (See Figure 4) is as follows:

(4) $$M_{c}\left(F\right)=\sigma \left(MLP\left(AvgPool\left(F\right)\right)+MLP\left(MaxPool\left(F\right)\right)\right)$$

Here, F is the input feature map, AvgPool is the global average pooling sum, MaxPool is the maximum pooling operation, MLP is the multilayer perceptron, and σ denotes the Sigmoid activation function.

#### 2.2.2. Spatial Attention Mechanisms

Spatial Attention Mechanism Module in CBAM: The output feature maps of the channel attention mechanism are processed in the spatial domain. First, they undergo maximum pooling and average pooling based on the channel dimension, and then the two output feature maps are stacked in the channel dimension layers to concatenate. Then, the number of channels is adjusted using 1 × 1 convolution, and finally, the weights are normalized using a sigmoid function. Lastly, the normalized weights are multiplied by the input feature degree [23].

**Figure 5:**
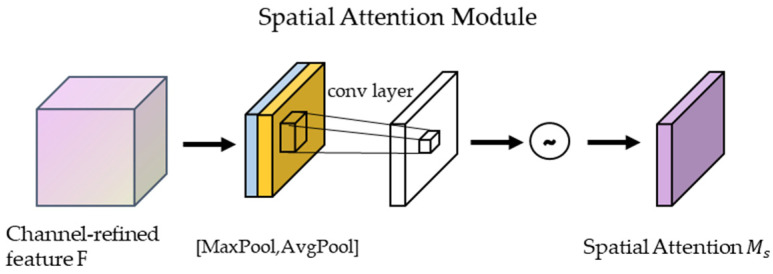
Spatial attention module.

The output of the spatial attention module (See Figure 5) $M_{s}\left(F\right)$ formula is calculated as follows:

(5) $$M_{s}(F)=\sigma (f^{\left(7\times 7\right)}([AvgPool(F);MaxPool(F)]))$$

$f^{7\times 7}$ is a 7 × 7 convolution operation, AvgPool(F) denotes average pooling, MaxPool denotes maximum pooling, and the resulting results are stitched together along the channel axis.

#### 2.2.3. Overall Workflow

The general process of CBAM: The input feature map is put through the channel attention mechanism. We then multiply the weights within the input feature map, and these are sent to the Spatial Attention Mechanism. We multiply the normalized weights with the values in the input feature map given by the Spatial Attention Mechanism to derive the final feature map [24]. This enhanced feature map takes other network structures as inputs to produce its network layer structures, retaining key information while reducing the effects of noise and removing redundant information.

### 2.3. Xception’s Network Structure

Xception is an improved model of Inception V3 proposed by Google, and its main improvement is the use of depth-wise separable convolution to replace the multi-size convolution kernel feature response operation used in the original Inception v3. The depth-wise separable convolution block consists of two parts: depth-wise convolution and 1 × 1 normal convolution [25]. The convolution kernel used for depth-wise convolution is usually 3 × 3 in size, making it a feature extraction operation, while 1 × 1 ordinary convolution is used to adjust the number of channels [26].

The purpose of the deep separable convolution block is to replace the normal 3 × 3 convolution approach with one with fewer parameters. Suppose there is a 3 × 3 convolutional layer with 16 input channels and 32 output channels. Here, the 3 × 3 convolutional kernels will traverse each of the 16 channels, producing 32 output channels requiring 16 × 32 × 3 × 3 = 4608 parameters. When applying deep separable convolution, 3 × 3 convolution kernels are used to traverse the data in each of the 16 channels to obtain 16 feature maps. Before the fusion operation, these 16 feature maps are then traversed with 1 × 1 convolution kernels, requiring 16 × 3 × 3 + 16 × 32 × 1 × 1 = 656 parameters. This shows that depth-separable convolution can significantly reduce the parameters of the model.

In layman’s terms, in the depth-separable convolution building block, a 3 × 3 convolution kernel is used to slide layer-by-layer over the input tensor; each convolution generates an output channel, and after the convolution is complete, the channel’s thickness is adjusted with 1 × 1 convolution [27].

**Figure 6:**
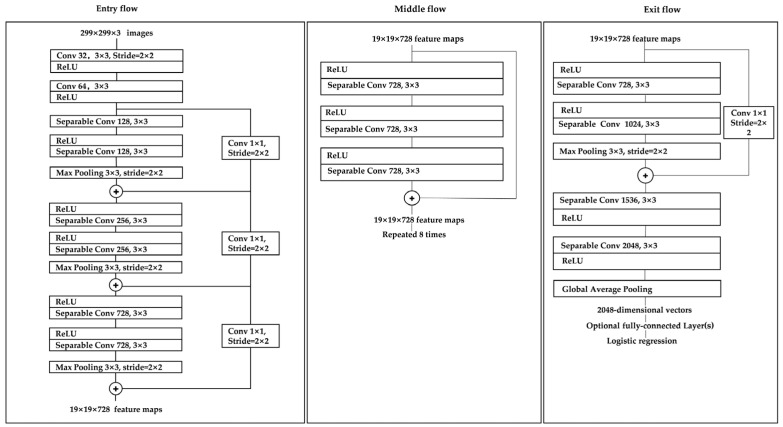
Xception Network Architecture.

The Xception model can be divided into three streams: Entry flow, middle flow, and exit flow, with a total of 14 blocks. The entry flow represents 4 blocks, the middle flow 8 blocks, and the exit flow 2 blocks. The specific structure is shown in Figure 6.

## 3. Motor Fault Diagnosis Method Based on Attention Mechanism and Lightweight Neural Network

The model collects vibration signals from a self-constructed experimental bench. These signals are then preprocessed, and Gram image coding is applied to encode time-domain information, transforming the one-dimensional vibration signals into grayscale maps to highlight their features. The lightweight neural network, Xception, is selected as the main tool, with the CBAM attention mechanism integrated into the model to enhance the importance of the characteristic information of motor faults, enabling accurate identification of these faults. The model was trained, validated, and tested on a Windows 11 64-bit operating system using PyCharm 3 January 2022, with an Intel^®^ Core™ i7-12800HX processor, NVIDIA GeForce 4070 GPU, and 16 GB of RAM. The hyperparameters were chosen as follows: a learning rate of 0.0001, a batch size of 32, the Adam optimizer, 20 epochs, and the cross-entropy loss function.

**Figure 7:**
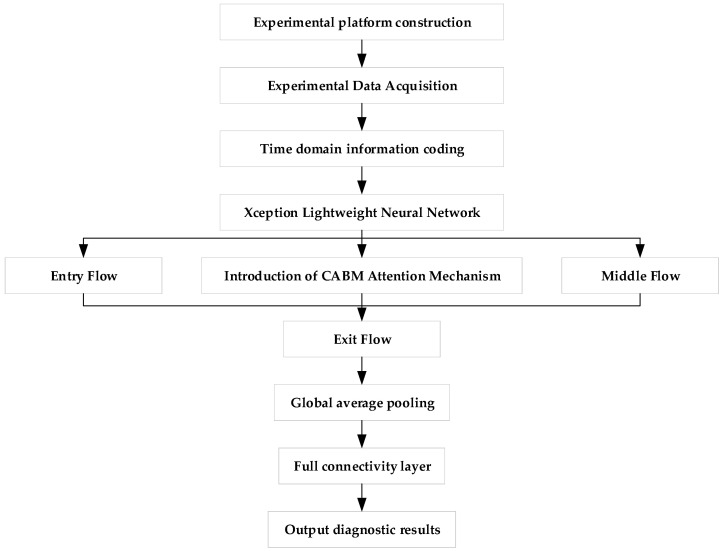
Fault diagnosis process.

In order to verify that the transformed Gram’s corner field map can be easily recognized, we entered the image into a lightweight neural network and added the CABM attention mechanism to the neural network. This lightweight neural network is based on the Inception network with deep convolution added, incorporating the idea of residual learning [28]. This structure enables the lightweight neural network to significantly reduce the influence of parameters under the premise of guaranteeing the classification and recognition ability of the model. The CABM attention mechanism is added so that the feature map input by the Gram’s corner field will continue after passing through the channel attention module and spatial attention module to pay attention to the most important features of motor failure, focus on important points in order to obtain more information from the features and identify the interference signals affecting the features, as well as useless signals. Interference signals that affect the features and useless information are ignored; this CABM attention mechanism greatly improves the efficiency and reliability of the processing of raw information. Therefore, we input the fused Gram angle field map into the CABM lightweight neural network such that the whole network can quickly and adaptively learn the different fault types that pertain to the motor, seize upon the most important features of motor faults, and analyze these features so as to classify different faults. This multi-model fusion method not only improves the rate of recognition of different faults but also minimizes the computational complexity and realizes the fast and accurate diagnosis of motor faults. The specific process followed by the model is shown in Figure 7.

The model is mainly composed of four parts: Entry flow, the CBAM attention mechanism, Middle flow, and Exit flow. Entry flow consists of two convolutional layers and three blocks. The kernel size of the convolutional layer is 3 × 3, the step size is 2, and the filling method and activation function are set to “same” and “Relu” [29]. Separable Convolution follows, followed by the activation function, then depth-wise separable convolution, and finally, the pooling layer. Depth-wise separable convolution starts with a 1 × 1 convolution kernel and then connects multiple 3 × 3 convolutions with a number of 3 × 3 convolutions equal to the number of output channels of the 1 × 1 convolution. Each block module is only different in terms of the number of convolution kernels; all other features are the same. CBAM starts from two avenues, channel and spatial, and introduces two analytical dimensions, spatial attention, and channel attention, to realize the sequential attention structure moving from channel to space. Spatial attention allows the neural network to pay more attention to the pixel regions in the image that play a decisive role in classification and to ignore the irrelevant regions, while channel attention is used to deal with the allocation relationship between feature map channels and the simultaneous allocation of attention to the two dimensions enhances the effect of the attention mechanism on the performance of the model [30].

The specific process of motor fault diagnosis based on CBAM–Xception is as follows:

1. Use the vibration sensor in the motor fault diagnosis experimental platform to collect the vibration and current signals of the motor, but not at the same time;
2. Convert these into separate Gram angle field diagrams to form a dataset, and randomly divide the dataset into a training set, a validation set, and a test set;
3. Construct the CBAM–Xception model and initialize the training parameters of the model;
4. Input the training samples into the CBAM–Xception model for training, and use the validation set to continuously optimize the model parameters until the completion of the iteration to obtain the trained model;
5. Input the test samples into the trained CBAM–Xception model to complete motor fault diagnosis and obtain the diagnosis results.

## 4. Experiment and Analysis of Motor Fault Diagnosis

### 4.1. Principle of Self-Driving Vehicle Fault Diagnosis Experiment Platform Construction

According to the “Autonomous Driving Safety First White Paper”, it is critical to verify and validate the safety of autonomous driving systems [31]. Testing and other activities should be conducted to ensure rigorous system design implementation and vehicle safety. Additionally, the literature indicates that the electric motor is the most important component in an electric vehicle, and it is used to convert electrical energy into mechanical energy. However, the risk of motor failures under various operating conditions increases with the operating time, affecting the reliability and safety of electric vehicles. Therefore, diagnosing the faults of electric motors in self-driving vehicles is highly meaningful. Tesla, as a representative manufacturer of electric vehicles, has begun to research and apply self-driving technology.

According to the information available regarding self-driving vehicles, onboard electric motors include AC motors and variable-frequency drive motors. In addition, the powertrain also includes the gearbox and braking system. Therefore, this study seeks to simulate the working state of a self-driving vehicle with an electric motor, variable-speed gearbox, magnetic powder brake, etc., and the detailed experimental platform is shown in Section 4.2.

### 4.2. Experimental Platform

**Figure 8:**
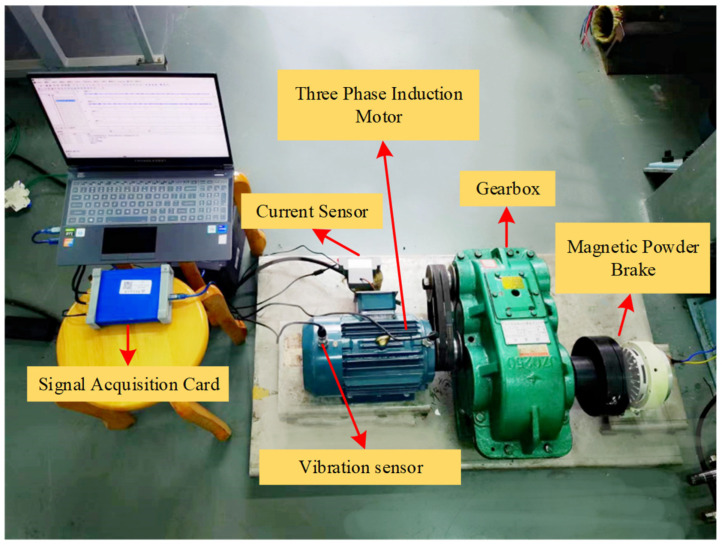
Experimental platform.

According to the building principle shown in Section 4.1, this paper designs an experimental platform for motor troubleshooting in self-driving vehicles, as shown in Figure 3. The experimental platform includes a three-phase asynchronous motor, a gearbox reducer, an inverter, and a magnetic powder brake used to simulate a real working environment. The vibration signal acquisition system consists of a YE6231 acquisition card, a CAYD051V acceleration sensor, and corresponding acquisition software. The three-phase asynchronous motor, gearbox, and magnetic powder brake are first fixed on the test bench. The three-phase asynchronous motor is connected to the power supply through an inverter. The motor and gearbox are connected with pulleys. During this experiment, vibration acceleration sensors were mounted at the drive and fan ends of the motor to capture the vibration signals. The experimental platform is shown in Figure 8.

The motor used in this experiment is a 3 kW three-phase induction motor, model YE2-100L-4, which meets the power requirements of the experimental platform. To collect motor signals under multiple working conditions, this study utilizes a frequency converter to adjust the speed by varying the current frequency. The inverter’s role in this experiment is solely to control the speed, with no special requirements; hence, the G7R5/P011-T4 model inverter is selected. The parameters of the three-phase induction motor, including rated power, rated voltage, rated speed, rated current, number of poles, and winding connection, are presented in Table 1.

**Figure 9:**
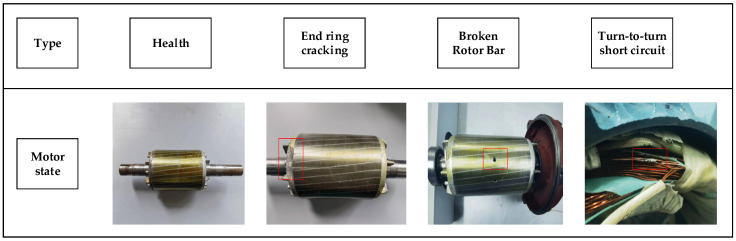
Motor Failure State.

Motor failure will have a huge impact on the smart driving car. Therefore, the fault diagnosis of electric motors is especially important. After analyses of many electric vehicle failures and using the information obtained by the U.S. Academy of Science and Technology, it has become known that the following types of motor failures occur during long-term operation: rotor breakage, turn-to-turn short-circuits, and ring breakage. These failures will cause abnormal vibration and temperature increases in the motor, which may induce other failures, and in severe cases, the motor may be damaged, causing downtime or life-threatening situations. Therefore, we established the above three cases to simulate real motor failures and chose different rotational speeds to collect vibration signals from a wide range of different failures. The failure of the rotor bar was induced to enable the artificial simulation of a fault hole; we also induced a short-circuit fault in the enameled wire using sandpaper to imitate the actual degree of failure, after which it was welded, cracking in the end ring was induced with a tool to simulate the destruction of the end ring. The experimental design included the operation of the three-phase asynchronous motor in four states (healthy, rotor bar break, turn-to-turn short-circuit, and end-ring crack) and at two operating speeds (30 Hz and 40 Hz), and the details of the faults are shown in Figure 9.

### 4.3. Experimental Data Description

The motor of the experimental platform was adjusted to 900 rpm and 1200 rpm, and the frequency converter was adjusted to the corresponding frequencies of 30 Hz and 40 Hz. A data acquisition card was used to collect continuous samples; the sampling frequency was set to 12,000 Hz, the sampling time was set to 8 s, and the sampling interval was set to 2 s. To collect vibration signals and current signals under different faults, the vibration signal sampling length was set to 640 k, and 800 samples of vibration signals and current signals were collected. The collection of 800 samples ensured that the two types of sensors could monitor all fault signals. The two types of sensors captured the following faults: cracked end ring, broken rotor bars, short circuits between gates, and degenerated motor health. After the collected data were processed, they were divided into separate scales, as presented in Table 2.

As can be seen from Table 2, the training set, validation set, and test set were randomly set up in the ratio of 5:2:3; there were 400 sets of training data, 160 sets of validation data, and 240 sets of test data for each single-sensor state. A total of 3200 sets of training samples, 1280 sets of validation samples, and 1920 sets of test samples were obtained for each of the eight states. The training set was composed of 1280 sets of validation samples and 1920 sets of test samples. The test set was composed of 1024 points. There were 1024 points in each sample; the training set constituted 3200 × 1024, the validation verification set constituted 1280 × 1024, and the test set constituted 1920 × 1024. Each sample was also converted into a Gram’s angle field map according to the cross-domain fusion method before training. The label codes corresponding to the motor states are shown in Table 3, and the category codes used in the confusion matrix analysis in this chapter and beyond follow this table.

**Figure 10:**
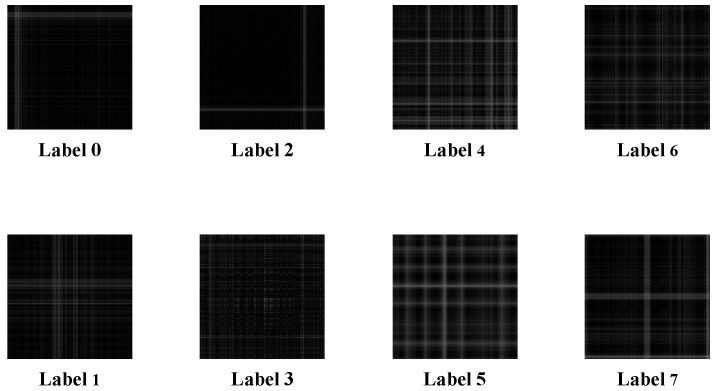
Plot of Gram’s angle field for each working condition.

The use of traditional one-dimensional data is more dependent on the faculty of human feature extraction, whereby expert experience is required to effectively extract data features and then identify and diagnose the type of fault. Motor faults can be found everywhere, and there are many types; when there is limited information, one-dimensional traditional data are affected by relatively large amounts of noise. In the case of complex faults, where one is unable to accurately identify the location or shape of the fault, pattern recognition is more difficult. In this situation, two-dimensional images show the superiority of using intuition. The Gram angle field, comprising a series of complex mathematical operations, features a solid mathematical foundation, and when there is a limited amount of data, it can be used to reduce the computational complexity of the data to a certain extent. The Gram angle field also has a high conversion rate, does not lose the characteristics of the motor fault, and can be in a number of fields. Therefore, this paper exploits the advantages of the Gram angle field by synthesizing one-dimensional data into a two-dimensional Gram angle field while also using the original vibration signals for time domain coding, thus greatly preserving the originality and authenticity of the data, resulting in eight very obvious motor fault characteristics in the map, which are easier to identify, as shown in Figure 10.

### 4.4. Performance Analysis of Fault Diagnosis Algorithms

The optimizer used for all models in this study is Adam, the loss mode is cross-entropy, and the initial value of learning is 0.0001. The final step of training is to set the number of iterations as 20 and to determine whether or not it is the optimal parameter via validation set loss. In addition, here, all networks were run under the deep learning framework TensorFlow 2.6.1 with hardware consisting of an 11th Gen Intel (R) Core (TM) i7-11800H @ 2.30 GHz, an Nvidia GeForce RTX 3070 GPU, and 16 GB RAM.

**Figure 11:**
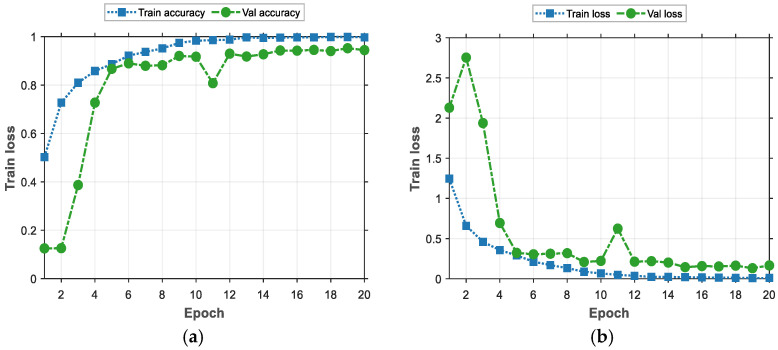
The training process of deep learning model. (**a**) Model accuracy. (**b**) Model loss value.

In order to better demonstrate the process of training the model, a line graph is used for visualization here, as shown in Figure 11.

The line graph provided in Figure 11 shows the performance of the model during training and validation. Firstly, in (a), based on the curve of Training Accuracy (Train Accuracy), we can see that the training accuracy shows a gradual improvement when there is a gradual increase in the training period. This indicates that the learning effect of the model on the training set gradually increases, and it can better adapt to the training data and more accurately capture the features of the data. Figure 11b demonstrates the training loss and validation loss that occurs with the procession of training rounds during the training process. Initially, the validation loss is high, at about 2.1, and then drops rapidly in the first few rounds of training, reaching about 0.28, after which it continues fluctuating around 0.1 in the subsequent rounds. This trend shows that the model’s performance, when applied to the validation set, improves with training and eventually stabilizes. It is worth noting that the values of the validation loss are consistently higher than those of training loss, which is normal because the model is optimized based on the training data during the training phase, while the validation loss is used to assess the model’s ability to generalize unseen data.

In summary, the trends of training and validation illustrate the learning and optimization effects of the model during the training process. As training proceeds, the model gradually learns the features of the data and achieves good generalization effects on the validation set, indicating that the model achieves better performance when applied to both the training and validation sets.

**Figure 12:**
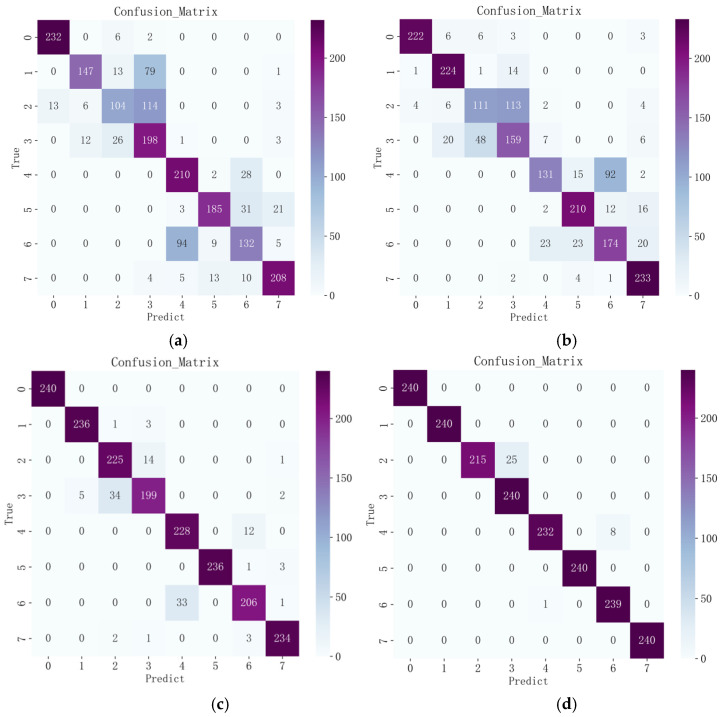
Model identification results. (**a**) Time Domain + CNN. (**b**) Time Domain+ ResNet. (**c**) Time Domain + Xception. (**d**) Time Domain + CBAM-Xception.

In order to demonstrate the superiority of the improved CBAM–Xception compared with the original model, this subsection uses convolutional neural networks and residual neural networks that have a similar structure to the lightweight neural network, as well as the unimproved lightened neural network for comparison. Convolutional neural networks are often used to process image data by using the internal convolutional layer and pooling layer, respectively, to extract features and reduce the dimensionality of the features before using the comprehensive class for classification prediction and fault diagnosis. The residual neural network represents an improvement on the convolutional neural network, incorporating residual blocks and thus solving the problem of gradient disappearance. It has a deeper network structure, better training effects, and superior performance, thus improving the performance of the deep network. Xception is the improved version of the neural net convolutional neural network, incorporating the concept of deep separable convolution with optimization. After testing by many scholars, its network performance has been shown to be very good, and its recognition and iteration speeds are also very fast. This article has undertaken an improvement of Xception; therefore, these three networks are chosen for comparison via experiments. The input set for all three networks was the image coding subjected to Gram’s corner field conversion to ensure equality in the experiment. In the following paper, the improved model will be further analyzed by focusing on the final recognition results. The confusion matrix was used here to visually compare the different models of CNN, ResNet, Xception, and CBAM–Xception after the Gram’s corner fielding of the original data. The recognition results are shown in Figure 12.

The confusion matrix illustrates the classification performance of the model in eight different categories (end ring cracked 30 Hz, end ring cracked 40 Hz, rotor broken bar 30 Hz, rotor broken bar 40 Hz, normal condition 30 Hz, normal condition 40 Hz, short turn 30 Hz, short turn 30 Hz, short turn 30 Hz), with an overall recognition rate of 98.23%. From the numbers on the diagonal, it can be concluded that the model exhibits high classification accuracy in most of the categories, which means that the model is able to accurately classify the samples into their correct categories. For example, the model showed high accuracy for four categories, label 0, label 1, label 5, and label 7, correctly classifying 240, 240, 240, and 240 samples, respectively.

However, we can observe some misclassification in the figures on the non-diagonal. In particular, there are some misclassifications in label 2 and label 4. These misclassifications are relatively small in number, but they may require further attention. They may stem from the similarity between categories or the effects of noisy data.

In Figure 12**,** we present the average classification results over ten runs for three different deep-learning models across eight motor states, providing insights into their performances in fault diagnosis by comparing their classification effects.

To validate the superiority of the proposed model, we compared each model and conducted ten training sessions. The average results are shown in Table 4. In Figure 12a, the classification accuracy of the CNN model on the whole is 73.80%. The large number of recognition errors for all working states may be due to the fact that the model encounters some difficulties in extracting features for that category. The model may need to develop deeper feature extraction capabilities for these categories in order to achieve better results. In Figure 12b, the overall classification accuracy of the ResNet model is 76.30%. There is an improvement compared to the CNN model, but the improvement is not significant. In Figure 12c, the Xception model performs well, with an overall classification accuracy of 93.96%. The accuracy is higher than those of (a) and (b) in all categories. Compared with the CNN and ResNet models, the improvement is 20.16% and 17.66%, respectively, which improvements are significant compared with the previous two models. From Figure 5, we can see that the accuracy of CBAM–Xception is 98.23%. Similar to the time-domain Xception model, the accuracy of this model is also higher than those of (a) and (b) for all categories, and the highest number of errors occurs in label 2, with a total of 25. This shows the model’s superior performance in handling this task. The introduction of the attentional mechanism also leads to an improvement in the accuracy of the Xception model.

Taken together, we see that the time-domain CBAM–Xception model performs well when applied to most fault types, with a stronger differentiation ability and better classification accuracy, especially when dealing with similar fault features and complex situations. It also achieves excellent performance in terms of training convergence and validation stability. This study can provide useful guidance for research and practical applications in the field of motor fault diagnosis and can help optimize the development of predictive maintenance strategies.

## 5. Conclusions

Focusing on the problem of the low accuracy of deep learning models when recognizing information pertaining to motor fault diagnosis under the complex working conditions of self-driving vehicles and the lack of rich single-domain data, this paper presents a comprehensive comparative analysis of four different deep learning models, including CNN, ResNet, Xception and CBAM–Xception. These models are also assessed in terms of time-domain data accuracy as well as cross-domain data accuracy, aiming to investigate their performance in the context of data generalization and practical applications.

The results of this paper show that the models achieve an impressive performance in the Gram’s corner field. In particular, for fault state classification, the cross-domain data + CBAM–Xception model exhibits excellent results in the zero-error classification of multiple categories within the confusion matrix. This highlights the high reliability and accuracy of the model when used in motor fault classification for self-driving vehicles, providing a solid foundation for practical applications.

In summary, the results of this paper emphasize the importance of the cross-domain data classification task to self-driving vehicle fault diagnosis and the excellent performance of the CBAM–Xception model in this task. Although this study has deeply analyzed the performance of cross-domain data and deep learning, there are still many directions worth exploring. These include model performance testing when more data have been captured, comparisons of different feature extraction methods, etc. The authors expect that such further studies will provide a wealth of further knowledge in the field of self-driving vehicle fault diagnosis and provide stronger support and guidance for engineering practice.

## Figures and Tables

**Table 1:** YE2-100L2-4 three-phase induction motor parameters.

Parameter Name	Numerical	Parameter Name	Numerical
Rated power/KW	3	Rated speed (r/min)	1430
Rated voltage/V	380	Polar logarithm	2
Rated current/A	6.78	Connecting method	Y

**Table 2:** Data presentation.

Number	Form	Number of Training Sets	Number of Validation Sets	Number of Test Sets
0	End ring cracking 30 Hz	400 × 1024	160 × 1024	240 × 1024
1	End ring cracking 40 Hz	400 × 1024	160 × 1024	240 × 1024
2	Broken Rotor Bar 30 Hz	400 × 1024	160 × 1024	240 × 1024
3	Broken Rotor Bar 40 Hz	400 × 1024	160 × 1024	240 × 1024
4	Health 30 Hz	400 × 1024	160 × 1024	240 × 1024
5	Health 40 Hz	400 × 1024	160 × 1024	240 × 1024
6	Turn-to-turn short circuit 30 Hz	400 × 1024	160 × 1024	240 × 1024
7	Turn-to-turn short circuit 30 Hz	400 × 1024	160 × 1024	240 × 1024

**Table 3:** Binary labeling codes.

Number	Category	Number	Category
0	End ring cracking 30 Hz	4	Health 30 Hz
1	End ring cracking 40 Hz	5	Health 40 Hz
2	Broken Rotor Bar 30 Hz	6	Turn-to-turn short circuit 30 Hz
3	Broken Rotor Bar 40 Hz	7	Turn-to-turn short circuit 30 Hz

**Table 4:** Ten times the average accuracy for each model.

Model	Accuracy
CNN	73.80%
ResNet	76.30%
Xception	93.96%
CBAM-Xception	98.23%

## Data Availability

The data used to support the findings of this study are available from the corresponding authors upon request.
